# Comparison of functional outcomes between single-radius and multi-radius femoral components in primary total knee arthroplasty: a meta-analysis of randomized controlled trials

**DOI:** 10.1186/s43019-020-00067-y

**Published:** 2020-10-02

**Authors:** Jahyung Kim, Kyung-Dae Min, Byung-Ill Lee, Jun-Bum Kim, Sai-Won Kwon, Dong-Il Chun, Yong-Beom Kim, Gi-Won Seo, Jeong Seok Lee, Suyeon Park, Hyung-Suk Choi

**Affiliations:** 1 Department of Orthopaedic Surgery, Soonchunhyang University Hospital Seoul, 59, Daesagwan-ro, Yongsan-gu, Seoul, 04401 South Korea; 2 Department of Orthopaedic Surgery, Soonchunhyang University Hospital Bucheon, Bucheon, South Korea; 3 Department of Orthopaedic Surgery, Smarton Hospital, Bucheon, South Korea; 4 Department of Orthopaedic Surgery, Soonchunhyang University Hospital Cheonan, Cheonan, South Korea; 5 Department of Orthopaedic Surgery, Soonchunhyang University Hospital Gumi, Gumi, South Korea; 6 Department of Biostatistics, Soonchunhyang University Seoul Hospital Seoul, Seoul, South Korea

**Keywords:** Single-radius, Multi-radius femoral component, Total knee arthroplasty, Meta-analysis, Functional outcome, Randomized controlled trial

## Abstract

**Purpose:**

Our purpose in the current meta-analysis was to compare the functional outcomes in patients who have received single-radius (SR) or multi-radius (MR) femoral components in randomized controlled trials (RCTs) for primary total knee arthroplasty (TKA). The hypothesis was that there would be no statistically significant difference between two groups in terms of functional outcomes.

**Materials and methods:**

We searched the international electronic databases PubMed, Embase, and the Cochrane Central Register of Controlled Trials up to February 2020 for RCTs that compared functional outcomes of SR and MR femoral component designs after primary TKA. We performed a meta-analysis of nine RCTs using the Knee Society Score for the knee (KSS-knee), KSS-function, Knee Injury and Osteoarthritis Outcome Score (KOOS), Oxford Knee Score (OKS), degree of knee flexion, extension, and complications, including postoperative infection and revision surgery.

**Results:**

The meta-analysis revealed no statistically significant differences in all the analyzed variables, including KSS-knee, KSS-function, KOOS, OKS, knee flexion, and knee extension. For postoperative complications, no statistically significant differences were detected for femoral component designs in postoperative infection or incidence of revision surgery between the two groups.

**Conclusions:**

The current meta-analysis of RCTs did not show any statistically significant differences between SR and MR femoral component designs in terms of postoperative functional outcomes. Evaluated outcomes included functional outcome scores, degree of knee flexion, extension, and complications. However, because of the limited clinical evidence of this study owing to the heterogeneity between the included RCTs, a careful approach should be made in order not to arrive at definite conclusions.

## Introduction

Considered to be one of the most successful procedures in the orthopedic field, total knee arthroplasty (TKA) has proven to be the gold standard treatment strategy for advanced symptomatic arthritis of the knee [1]. In an effort to increase the patient satisfaction and reproduce the biomechanics of a natural knee joint, surgeons and engineers have developed a variety of surgical techniques, advanced prosthesis designs, and refined implantation strategies in TKA [2, 3]. The long-term clinical results of TKA have, thus, been advantageous in terms of implant survival rate, reported to be above 90–95% over 10–15 years [4, 5].

However, the aforementioned success of TKA does not always represent the subjective functional impression of each patient, since several studies have reported dissatisfaction rates of up to 25% [6, 7]. Among the multi-factorial causes responsible for dissatisfaction or compromised functional consequences, abnormal kinematics of TKA systems compared to natural knees could be a significant factor affecting either instability or decreased extensor moment arms after the surgery [8, 9].

In this respect, comparison of single-radius (SR) or multi-radius (MR) femoral component designs is an interesting topic in the literature. The so-called MR femoral component was historically first introduced in 1971 and is still widely used [10]. It was designed to reproduce the then-accepted J-shaped radius of the natural femoral condyle curvature [11]. However, as TKA came to be offered to a younger population, higher clinical expectations among patients produced a variety of postoperative dissatisfactions. In line with increased interest in new designs to promote better clinical results, the concept of SR femoral components came to the fore [12]. Because of the single radius of the femoral condyle curvature, the SR femoral component design may provide more posterior and distal axes than does the conventional MR design. As a consequence, longer extensor moment arms to improve mechanical efficacy and maintain equidistant conversion over the entire range of motion (ROM) could be achieved in SR femoral components from a mechanical aspect [13].

Despite the theoretical advantages of SR, it is still debatable whether the advantages of the SR femoral component design provides better functional outcomes than does the MR femoral component design in practice [13–15]. In an effort to investigate this unexplained issue, Liu et al. [16] recently did a meta-analysis comparing SR with MR and concluded that no significantly preferable functional outcomes could be detected between the two designs. However, it was biased by the limited overall quality because of its inclusion of observational studies with only seven randomized controlled trials (RCTs).

Because of the increased amount of high-level literature, summarizing and analyzing the level 1 evidence studies focused on comparing the functional outcomes after TKA based on the distinct femoral designs is considered valuable. Therefore, our aim in the present study was to do a meta-analysis of RCTs to find out whether there are differences in postoperative functional outcomes between SR and MR femoral components in TKA. The hypothesis was that there would be no statistically significant differences between the two groups in their functional outcomes.

## Materials and methods

### Data source and search strategy

This study is based on the Cochrane Review Methods [17]. We thoroughly searched PubMed, Embase, the Cochrane Central Register of Controlled Trials, and the Web of Science up to February 2020. The free text including these terms was searched: “(single radius OR multi-radius OR constant radius OR Triathlon OR Scorpio OR NRG) AND (total knee arthroplasty OR total knee replacement).” The references cited by the retrieved articles and bibliographies of other relevant studies were also screened manually for any additional trials. In order to cover the possibly missed trials, we used additional strategies as follows: (1) after the initial electronic search, we hand-searched further relevant articles and the bibliographies from identified studies; (2) we also did specific searches in terms of diverse prosthetic systems, known to be SR femoral components, by using their brand names.

### Study selection

All studies to be included were independently chosen by two reviewers based on the selection criteria. Study selection was made through two levels of screening. At the first level, we screened titles and abstracts of identified studies, followed by the second level of screening using the full text. Studies were included in our meta-analysis if they met these criteria: (1) patients with knee arthritis (osteoarthritis, rheumatoid arthritis, or post-traumatic osteoarthritis) or osteonecrosis and presenting for primary TKA; (2) TKA intervention in which a SR prosthesis was compared with a MR prosthesis. The prostheses were cemented, and fixed bearing, and there were no restrictions about posterior-cruciate retaining/substituting or patellar resurfacing; (3) outcome measures included a method of functional assessment, such as Knee Society Score (KSS) or Knee Injury and Osteoarthritis Outcome Score (KOOS); and (4) prospective RCTs. The language was restricted to English. Whenever the outcomes of a trial were reported at multiple follow-up periods, we collected the data into different subgroups of similar periods. Articles were excluded from our study if: (1) patients presented with a primary surgical intervention of the knee other than TKA or revised TKA; (2) no comparison between SR and MR was done; (3) prostheses were non-cemented or mobile bearing; (4) functional outcomes were not reported; and (5) study designs other than RCTs were used, such as non-randomized, duplicated studies, animal or cadaver studies, biomechanical studies, observational studies, case reports, letters to the editor, correspondence, and review articles.

### Data extraction

Data were extracted separately by two reviewers from each study using a predefined extraction form. Any disagreement unresolved by discussion was put under the review of a third author. The following variables were extracted from studies: (1) mean and standard deviation (SD) or total event of the parameters of SR and MR groups; (2) demographic characteristics (e.g., number of patients, brand of prosthesis, gender ratio, average age, and mean follow-up periods); (3) method of functional outcome assessment; and (4) follow-up period after the surgery.

If the above variables were not mentioned in the studies, we asked for the data via e-mail.

### Assessment of methodological quality

**Fig. 1:**
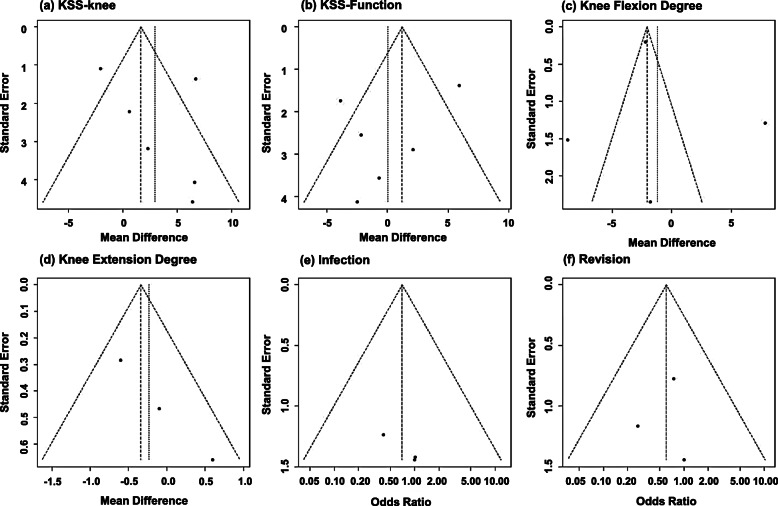
Funnel plot for publication bias

Two researchers independently selected all articles following the above selection criteria while assessing the quality of selected articles. Any disagreement was resolved through discussion with the corresponding researcher. The Physiotherapy Evidence Database (PEDro) scale was used for RCTs. The scale comprised 11 items based on the Delphi list to assess the methodological quality of each article. To assess the publication bias, we used Begg’s rank test and a funnel plot among variables comparing more than two studies (Fig. 1). There was no evidence of significant publication bias (*p* = 0.348 for KSS-knee; *p* = 0.851 for KSS-function; *p* = 1 for knee-flexion angle; *p* = 0.117 for knee-extension angle; and *p* = 0.602 for both infection and revision).

### Statistical analysis

The R version 3.3.1 (“meta” packages) and Rex software (https://rexsoft.org/) were used for the meta-analyses. The main parameters of our review were function assessment systems (e.g., KSS, KOOS, and OKS), knee flexion (degrees), and knee extension (degrees), which were described as continuous data. In addition, dichotomous data of complication and revision were evaluated. Q-statistics tests and I^2^ were used for evaluating heterogeneity among studies [18]. A random-effects model with the DerSimonian-Laird method was used for all data analysis because of the small study numbers. For estimating the variance component, the Mantel-Haenszel (M-H) method was used for dichotomous data and the inverse variance method was used for continuous data. The weighted mean difference (WMD) and 95% confidence interval (CI) were calculated for continuous data, and the odds ratio (OR) and 95% CI for SR compared with MR were calculated for dichotomous data.

## Results

**Fig. 2:**
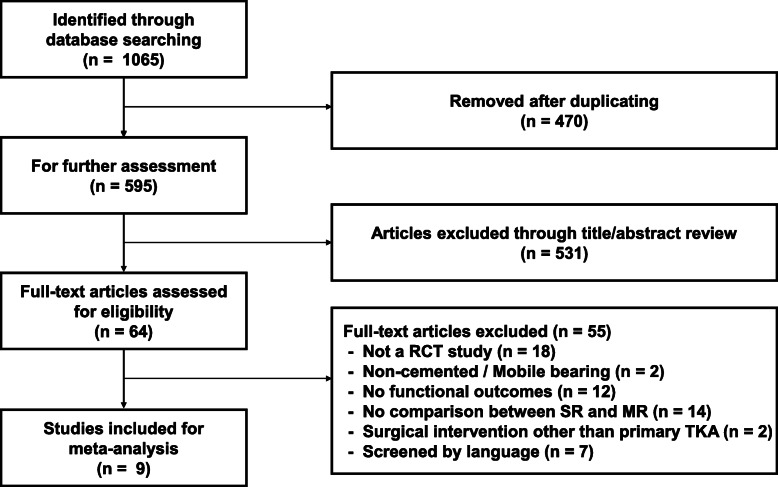
Flowchart showing details of the literature search

We identified 595 articles initially through both online and manual searches undertaken up to February 2020. After we applied the exclusion criteria, ultimately nine RCTs remained to be analyzed in this meta-analysis [14, 19–26] (Fig. 2). Characteristics and demographic data of the included studies are provided in Table 1. In terms of the quality of each study, trials with PEDro scores ≥ 6 were considered to be of high quality, and all the RCTs showed scoring of high quality (Table 2). In total, 938 patients were enrolled in the meta-analysis. In total, 475 patients had received the SR prosthesis, and 463 patients had received the MR prosthesis.

**Table 1:** Characteristics and demographic data of included randomized controlled trials

Study	Year	Country	Number of patients	Number of knees	CR/PS	Brand of prosthesis (SR: MR)	SR	MR	Gender (M/F)		Average age (y)		Mean follow-up (mo)
									SR	MR	SR	MR	
Hall [14]	2008	USA	100	100	CR	Scorpio: PFC	50	50	n/s	n/s	69.5	72.6	12
Molt [24]	2012	Sweden	60	60	CR	Triathlon: Duracon	30	30	8/22	13/17	69	66	24
Jo [21]	2014	South Korea	100	100	CR	Scorpio: NexGen	50	50	6/44	9/41	66.4	67.5	36.8
Hamilton [20]	2015	UK	212	212	CR	Triathlon: Kinemax	108	104	46/62	35/69	69.3	68.8	36
Kim [22]	2015	South Korea	120	120	PS	Triathlon: PFC Sigma	60	60	n/s	n/s	67.2	67.2	12
Larsen [23]	2015	USA	32	32	PS	Triathlon: Vanguard	16	16	8/8	8/8	71.6	70.9	12
Collados-Maestre [19]	2017	Spain	237	237	CR	Trekking: Multigen	118	119	37/81	35/84	71.9	10.6	68.4
Wellman [26]	2017	USA	40	40	CR	Triathlon: NexGen	20	20	8/12	10/10	61.9	63.1	12
Mushtaq [25]	2018	UK	105	105	PS	Scorpio: AGC	51	54	n/s	n/s	n/s	n/s	12

*SR* single radius, *MR* multiple radius, *M* male, *F* female, *n/s* not stated, *mo* month, *CR* cruciate-ligament-retaining, *PS* posterior-cruciate-ligament-stabilized

**Table 2:** Physiotherapy Evidence Database (PEDro) critical appraisal tool results of 9 randomized controlled trials

Study		PEDro criteria											Total
		1	2	3	4	5	6	7	8	9	10	11	
Hall [14]	2008	Y	Y	N	Y	N	N	Y	Y	Y	Y	Y	8
Molt [24]	2012	Y	Y	Y	Y	Y	N	N	Y	Y	Y	Y	9
Jo [21]	2014	Y	Y	Y	Y	N	N	N	Y	Y	Y	Y	8
Hamilton [31]	2015	Y	Y	N	Y	Y	N	Y	Y	Y	Y	Y	9
Kim [22]	2015	Y	Y	N	Y	N	N	Y	Y	Y	Y	Y	8
Larsen [23]	2015	Y	Y	N	Y	N	N	N	Y	Y	Y	Y	7
Collados-Maestre [19]	2017	Y	Y	N	Y	N	Y	Y	Y	Y	Y	Y	9
Wellman [26]	2017	Y	Y	N	Y	N	N	N	N	Y	Y	Y	6
Mushtaq [25]	2018	Y	Y	Y	Y	Y	N	Y	Y	Y	Y	Y	10

*PEDro* Physiotherapy Evidence Database scale, *RCT* randomized controlled trial, *Y* yes, *N* no. Criteria: 1. eligibility criteria were specified; 2. subjects were randomly allocated to groups (in a crossover study, subjects were randomly allocated in the order in which treatments were received); 3. allocation was concealed; 4. the groups were similar at baseline in the most important prognostic indicators; 5. there was blinding of all subjects; 6. there was blinding of all therapists who administered the therapy; 7. there was blinding of all assessors who measured at least one key outcome; 8. measures of at least one key outcome were obtained from more than 85% of the subjects initially allocated to groups; 9. all subjects for whom outcome measures were available received the treatment or control condition as allocated or, where this was not the case, data for at least one key outcome was analyzed by “intention to treat”; 10. the results of between-group statistical comparisons are reported for at least one key outcome; 11. the study provides both point measures and measures of variability for at least one key outcome

There were seven functional assessment systems in the included articles: the Knee Society Score (KSS), the Western Ontario and McMaster Universities Osteoarthritis Index (WOMAC), the Hospital for Special Surgery (HSS) Knee Score, the Visual Analog Scale (VAS), the Knee Injury and Osteoarthritis Outcome Score (KOOS), the International Knee Society Score, the Oxford Knee Score (OKS), the University of California at Los Angeles (UCLA) Knee Score, and the Lower Extremity Activity Scale (LEAS) score. However, only the KSS, the KOOS, and the OKS were included as a functional assessment score in this meta-analysis because there were no overlapping studies in terms of other score system; so they were not eligible for comparison between studies. Similarly, although five types of complications were assessed in all the studies (infection, aseptic loosening, stiffness, anterior knee pain, revision surgery), only the incidence of postoperative infection and revision surgery could be used to compare between the SR and MR prostheses. Furthermore, the knee-flexion degree and knee-extension degree were analyzed.

### KSS-knee

**Fig. 3:**
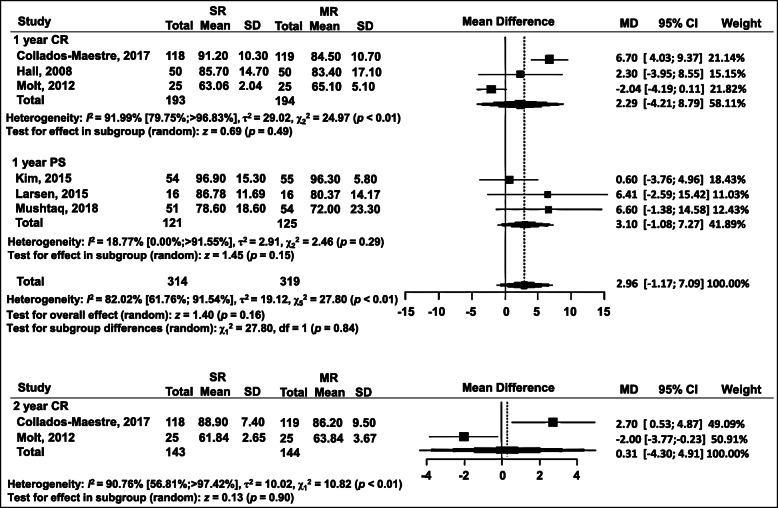
Forest plot of meta-analysis: Knee Society Score for the knee (KSS)-knee

The KSS-knee score was used in six RTCs. We divided the KSS-knee results into two subgroups based on the follow-up period of 1 or 2 years, which showed no significant difference between any of the subgroups (Fig. 3). One-year follow-ups were again divided in terms of prosthesis design: the posterior-cruciate-ligament-retaining (CR) subgroup contained three studies (WMD = 2.29, 95% CI = − 4.21 to 8.79 (*p* = 0.49)); the posterior-cruciate-stabilized (PS) subgroup contained three studies (WMD = 3.10, 95% CI = − 1.08 to 7.27 (*p* = 0.15)); total (WMD = 2.96, 95% CI = − 1.17 to 7.09 (*p* = 0.84)) (Fig. 3). Two-year follow-ups were included in two studies: WMD = 0.31, 95% CI = − 4.30 to 4.91 (*p* = 0.90) (Fig. 3).

### KSS-function

**Fig. 4:**
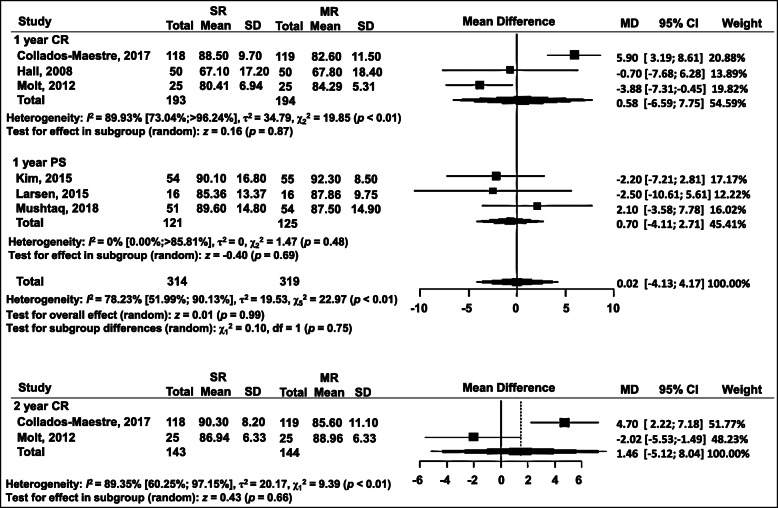
Forest plot of meta-analysis: Knee Society Score for the knee (KSS)-function

Six studies used this outcome measure. The same type of subgroup division was used as in KSS-knee, and no significant difference was detected in KSS-function either (Fig. 4). One-year follow-ups were divided in terms of prosthesis design: the CR subgroup contained three studies (WMD = 0.58, 95% CI = − 6.59 to 7.75 (*p* = 0.87)); the PS subgroup contained three studies (WMD = − 0.70, 95% CI = − 4.11 to 2.71 (*p* = 0.69)); total (WMD = 0.02, 95% CI = − 4.11 to 2.71 (*p* = 0.48)) (Fig. 4). Two-year follow-ups were included in two studies: WMD = 1.46, 95% CI = − 5.12 to 8.04 (*p* = 0.66) (Fig. 4).

### KOOS

**Fig. 5:**
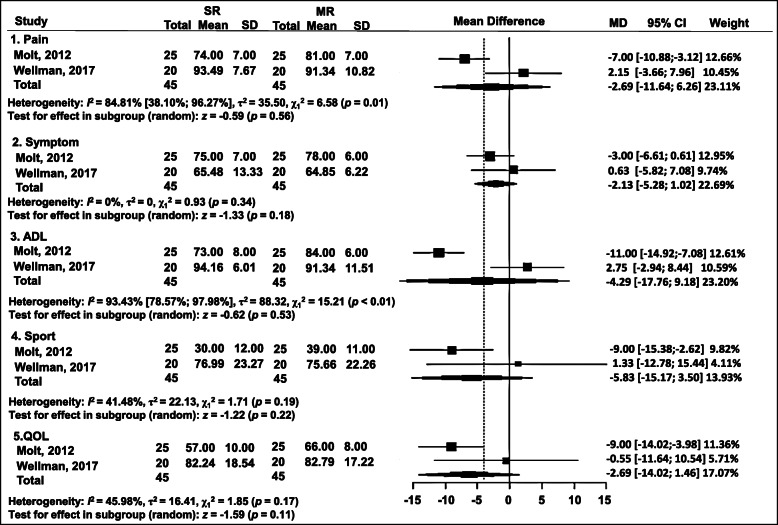
Forest plot of meta-analysis: Knee Injury and Osteoarthritis Outcome Score (KOOS)

This outcome assessment was available in two RTCs at 1 year postoperatively (Fig. 5). No significant differences were detected for the parameters of the subscores: (1) pain: WMD = − 2.69; 95% CI = − 11.64 to 6.26 (*p* = 0.56); (2) symptoms: WMD = − 2.13; 95% CI = − 5.28 to 1.02 (*p* = 0.18); (3) activities of daily life (ADL): WMD = − 4.29; 95% CI = − 17.76 to 9.18 (*p* = 0.53); (4) sports: WMD = − 5.83; 95% CI = − 15.17 to 3.50 (*p* = 0.22); and (5) quality of life (QOL): WMD = − 6.28; 95% CI = − 14.02 to 1.46 (*p =* 0.11) (Fig. 5).

### OKS

**Fig. 6:**
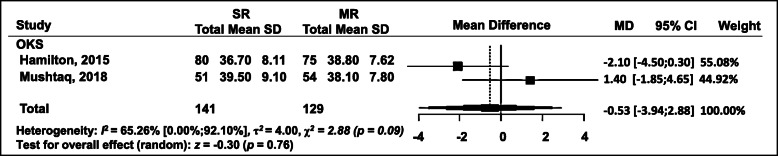
Forest plot of meta-analysis: Oxford Knee Score (OKS)

This outcome assessment was available in two studies at 1 year postoperatively (Fig. 5). The differences were not statistically significant in the meta-analysis: WMD = − 0.53; 95% CI = − 3.94 to 2.88 (*p* = 0.76) (Fig. 6).

### Range of motion

**Fig. 7:**
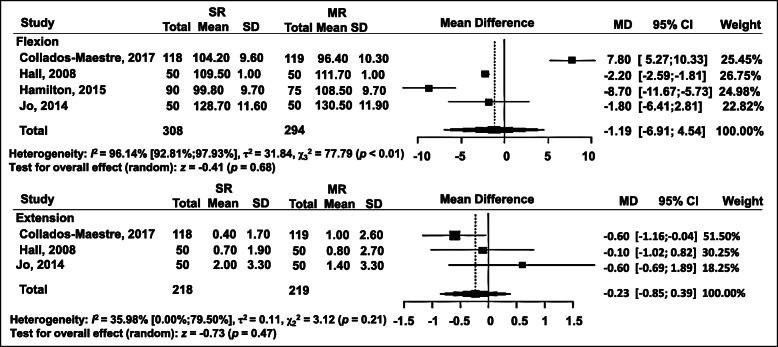
Forest plot of meta-analysis: flexion, extension

In terms of ROM, postoperative knee-flexion degree and knee-extension degree were used to compare the SR and MR groups (Fig. 7). Postoperative knee flexion was assessed in four RCTs, without significant differences detected between the SR and MR groups: WMD = − 1.19; 95% CI = − 6.91 to 4.54 (*p* = 0.68). In addition, postoperative knee extension was evaluated in three studies, also revealing no significant difference: WMD = − 0.23; 95% CI = − 0.85 to 0.39 (*p* = 0.47) (Fig. 7).

### Complications

**Fig. 8:**
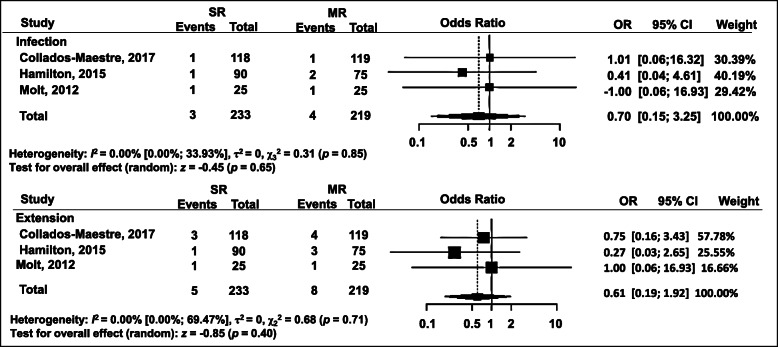
Forest plot of meta-analysis: complications

Three studies assessed the incidence of postoperative infections, with three out of 233 knees (1.3%) in the SR group and four out of 219 knees (1.8%) in the MR group. No significant difference was detected between the two groups: OR = 0.70; 95% CI = 0.15 to 3.25 (*p* = 0.65) (Fig. 8). For revision surgery after primary TKA, data were collected from the three studies with five of the 233 patients (2.1%) in the SR group and eight of the 219 (3.7%) in the MR group. However, no significant difference was detected between the two groups: OR = 0.61; 95% CI = 0.19 to 1.92 (*p* = 0.40) (Fig. 8).

## Discussion

Despite the reasonable radiologic findings and functional outcomes following TKA, some patients are still not satisfied and complain of less optimal functional outcomes than expected. Noble et al. [27] reported that 52% of patients reported limitations in functional activities, because TKA does not restore normal knee function, independent of the effects of age and gender. All kinds of different functional limitation include kneeling, squatting, lateral movement, turning and cutting, carrying loads, stretching, leg-strengthening, tennis, dancing, and gardening. Much of these differences are reflections of the biomechanical deficiencies of contemporary femoral component designs, which eventually leads to the necessity for improvements in the surgical procedure and the prosthetic designs. Although some studies have found excellence between both the SR and MR femoral components in TKA, the results are still unclear and are insufficient for quantitative analysis comparing functional outcomes between the SR femoral component and the MR femoral component [28]. Therefore, we aimed to compare the functional outcome differences including complications between the SR and MR femoral component groups focusing on RCTs for primary TKA.

The key finding of our meta-analysis of nine RCTs was that the SR femoral component group showed no statistically significant differences from the MR femoral component group in primary TKA in terms of functional outcome parameters. In addition, no significant difference could be observed in terms of incidence of postoperative complications or revision surgery. These findings were comparable to those of a previous meta-analysis done by Liu et al. [16] among seven RCTs and eight observational studies. However, since the clinical relevance of the results from this meta-analysis is limited owing to relatively small number of the included studies, these findings should be interpreted carefully.

Theoretically, a SR femoral component puts the posterior flexion-extension axis backward, which results in lengthening of the extensor moment arm. Such alteration of a SR femoral component is known to effectively reduce both quadriceps muscle force and joint reaction force [29]. In addition, the concept of midrange flexion instability increased the belief that the risk of instability might increase in MR design, whose radius was not constant during knee flexion [30]. In line with such advantages, Collados-Maestre et al. [19] reported that the SR femoral component provided significantly better functional outcomes than did the MR component in a randomized trial comparing 118 SR with 119 MR femoral designs in a 5.7-year follow-up. Additionally, Hamilton et al. [31] demonstrated that, at a mean of 8.12 years’ follow-up, the SR group was statistically better than the MR group in terms of knee flexion, lower-limb power output, and report of worst daily pain experienced. Furthermore, Luo et al. [32] found less anterior knee pain and painless crepitation among the SR group in the long-term follow-up study of a minimum of 10 years. These studies are supported by the fact that better functional outcomes of SR have been observed in many of the large study groups with long-term follow-up period [33, 34].

In contrast, others ask whether these theoretical benefits of SR designs actually correlate with superior postoperative functional outcomes. Based on the cadaveric studies comparing kinematics and stability for femoral component designs, Stoddard et al. [35] found no differences in instability for any degrees of flexion. They insisted that the shape of the femoral condyle would not directly contribute to the midrange flexion instability. In addition, Kim et al. [22] assessed the extent of quadriceps’ strength recovery following primary TKA using a dynamometer and found no notable difference in relation to the radius design of the femoral component. These findings correlated with the results of the randomized studies that focused on the postoperative functional outcomes. Mushtaq et al. [25] compared 54 patients with MR and 51 with SR and observed no significant differences in KSS and OKS. Moreover, Jo et al. [21] compared 50 patients with SR and 50 with MR, and found no significant differences in HSS score, ROM, and WOMAC either. Similarly, Hall et al. [14] compared 50 patients in both the SR and MR groups, and detected no significant differences in postoperative KSS, flexion, and knee extension. These findings, which correlate with the results of our present meta-analysis, suggest that it would not be adequate to conclude that the functional outcome of TKA is solely dependent on the difference of the radius of the femoral component rather than on a combination of various other factors including preoperative education, surgical technique, or postoperative rehabilitation [35–37].

In order to quantify the postoperative functional outcome, we used a string of functional assessment scoring systems. However, current scoring systems to describe functional outcomes after TKA are not sufficient to reflect the actual functional outcomes thoroughly for many reasons. First, most of these systems have a ceiling effect, and so have difficulty in discriminating the delicate changes at the high functional levels [38, 39]. Second, patients and physicians evaluate the results differently, because physicians are more likely to be satisfied than patients with TKA results [40]. In other words, caution should be taken when generalizing the physician outcome scores (e.g., Knee Society Score) to the patient self-assessed outcomes (e.g. Oxford Knee Score) [41]. In addition, patient-reported functional outcomes may be related to the pain or function of the contra-lateral limb, inhibiting the accurate measurement of functional outcome in the involved limb [42]. Lastly, factors like cognitive function, socioeconomic, or psychological status can influence the patient self-reports [43–45]. Therefore, it would be appropriate to evaluate the postoperative functional outcomes by combining the performance-based measurement, clinician-assessed outcome, and patient-perceived outcomes together.

Recently, Li et al. [46] performed a meta-analysis of RCTs and proposed that a SR design may result in improved range of movement (ROM) and better extensor function, ending up with distinct conclusions compared with previously performed meta-analyses that included observational studies [16]. However, their meta-analysis had some serious methodological errors. Of the 12 studies included, three of them did not fulfill the inclusion criteria of the study. First, a study by Menciere et al. [47] is a retrospective comparative case-control study rather than a prospective RCT. Second, a study by Tamaki et al. [48] is focused on the impingement of the anterior tibial post while walking in the femoral component design, which included 20 patients who underwent successful TKA, resulting in over 90 points of the KSS score, instead of native knees. Lastly, a study by Schmitt et al. [49] is intended to assess the outcome of navigated TKA in comparison with conventional implantation, and the TKA of both the SR and the MR groups was done under navigation. Since the surgical procedure differed from that in the other RCTs included in the meta-analysis, it would be proper to exclude such a study. Therefore, it would be appropriate to interpret that Li et al. have drawn a biased conclusion without a high level of evidence.

### Limitations

Our current meta-analysis has some limitations that should be taken into account. First, functional outcomes used in trials could not directly support all the differences between the SR and MR groups because there were few RCTs included in this study, and mutually exclusive scoring systems were used in the studies. These limitations precluded not only further analysis upon the known, different variables, but also detailed subgroup analysis regarding the follow-up period or other issues of femoral component designs like CR or PS. Thus, precautions are required when interpreting these results. Second, although we assessed the quality of RCTs using a quality assessment tool and found that all of them had good quality clarification, we could not evaluate the methodological quality for each study impeccably. Third, the follow-up periods of all the included trials were relatively short, with a maximum of 5 years. Consequently, the results of this study may lead to different conclusions with longer follow-up periods. Last, there was heterogeneity between RCTs in demographics, type of prostheses, surgical techniques, and postoperative rehabilitation programs, which might have affected the outcomes. Therefore, a succeeding study would have to make an effort to minimize the heterogeneity between the included studies.

## Conclusion

The current meta-analysis of RCTs did not show any statistically significant differences between SR and MR femoral component designs in terms of postoperative functional outcomes. Evaluated outcomes included functional outcome scores, degree of knee flexion, extension, and complications. However, because of the limited clinical evidence of this study owing to the heterogeneity between the included RCTs, a careful approach should be made in order not to arrive at definite conclusions.

## Data Availability

All data generated or analyzed during this study are included in this published data.

## Abbreviations

CI: Confidence intervals
CR: Cruciate-ligament retaining
PEDro: Physiotherapy Evidence Database
PS: Posterior-cruciate stabilized
KOOS: Knee Injury and Osteoarthritis Outcome Score
KSS: Knee Society Score
MR: Multi-radius
OKS: Oxford Knee Score
OR: Odds ratio
RCTs: Randomized controlled trials
SR: Single-radius
TKA: Total knee arthroplasty
WMD: Weighted mean difference
